# Health issues of concern to Africa and beyond: infections, NCDs and health systems inertia

**DOI:** 10.4314/ahs.v18i1.1

**Published:** 2018-03

**Authors:** James K Tumwine

In this March 2018 issue, we bring you papers on infections, non-communicable diseases and sexual/reproductive health.

## Infections

The issue begins with an interesting paper on silencing a nosocomial pathogen, using glyceryl trinitrate. The authors contend that glyceryl trinitrate is “a quorum sensing and virulence indicator”- and that this might have potential for treating nosocomial infections.^1^

The same authors report alarming rates of antibiotic resitance by *Pseudomonas aeruginosa* isolated from patients with urinary tract infections in Zagazig, Egypt.^2^ We have other infectious disease papers- including brucellosis in Uganda^3^, and leprosy in Indonesia.^4^

The ubiquitous cockroach is back in the news! Researchers from Jimma in Ethiopia have identified multi-drug resistant food borne illness associated with bacteria isolated from cockroaches in “eating” places.^5^

From Tunisia, we have two papers on mosquitoes resistant to available insecticides, a cause for concern!^6^,^7^

The West African Ebola epidemic is long over, but it continues especially in Nigeria.^8^

From Northern Tanzania, we have an interesting paper on esophageal candidiasis among patients in the endoscopy unit in Bugando, Mwanza.^9^ From Nigeria, Babajide and others report on their study on knowledge awareness and practice of infection control.^10^

This section ends with a treatise on human bite, an ageold weapon of assault in African societies!^11^

## Non-communicable diseases

While this is a diverse category, NCDs, as they are more popularly known are in ascendency.

Egyptian pulmonologists report on high-resolution computed tomography (HRCT) as a tool for evaluation of COPD.^12^ While smoking is clearly deleterious to health, its implication among mentally ill patients has not been established.^13^

Saudi scientists bring us two papers on the impact of aerobic exercises on inflammatory markers among patients with sickle cell anaemia^14^ and the elderly.^15^

Illicit drug use has reached epidemic levels in Africa, and South Africa has not been spared. Wicomb and others bring us an interesting paper on illicit drug use and violence in acute psychosis.^16^

Keeping with the mental health theme, we bring you a paper on early diagnosis and intervention for autism spectrum disorder.^17^ The NCD section ends with a case report on Truncus arteriosus communis.^18^

## Sexual and reproductive health (SRH)

This section begins with a Gambian study on the outcome of caesarean section and deliveries over a one year period^19^, and it is followed by a Ugandan experience of teaching health workers obstetrics ultrasound.^20^ Continuing with the teaching theme in SRH, Turkish researchers report on their experience with fertility and expectations of having children.^21^

We have a disturbing paper from Ibadan Nigeria on risk factors for sexually transmitted infections of street youths, where the majority are sexually active.^22^

The treatise ends with papers on health extension workers in Ethiopia^23^, and a letter on responsible conduct of research.^24^

All in all, the issue shows serious quantitative and qualitative research into issues affecting the health of people of Africa and beyond.
